# Protocol for the Flinders Kidney Health Registry: patient outcomes of kidney cancers and nephrectomies

**DOI:** 10.1186/s12894-022-01065-w

**Published:** 2022-07-21

**Authors:** Jordan Y. Li, Sarah Bodda, Alex Jay, Ganessan Kichenadasse, Michael Chong, Jonathan M. Gleadle, Michael O’Callaghan

**Affiliations:** 1 Department of Renal Medicine, Flinders Medical Centre, Flinders Drive, Bedford Park, Adelaide, SA 5042 Australia; 2 Department of Urology, Flinders Medical Centre, Flinders Drive, Bedford Park, Adelaide, SA 5042 Australia; 3 Department of Medical Oncology, Flinders Medical Centre, Flinders Drive, Bedford Park, Adelaide, SA 5042 Australia; 4 Flinders Health and Medical Research Institute (FHMRI), College of Medicine and Public Health, Flinders University, Adelaide, SA 5042 Australia

**Keywords:** Ablation, Chronic kidney disease, Creatinine, End stage kidney disease, Metastasis, Nephrectomy, Registry, Renal cell carcinoma, Small renal mass, Surveillance

## Abstract

**Background:**

Kidney cancer accounts for 2% of new cancers diagnosed in Australia annually. Partial and radical nephrectomy are the treatment of choice for kidney cancer. Nephrectomy is also performed for living donor kidney transplantation. Nephrectomy is a risk factor for new-onset chronic kidney disease (CKD) or deterioration of pre-existing CKD. Understanding the risk factors for new-onset or deterioration of existing CKD after nephrectomy is important in developing preventive measures to provide better care for these patients. There is also a need to understand the incidence, natural history, management trends, and sequelae of radiofrequency ablation as well as surveillance of small renal cancers or small renal masses (SRMs). Clinical registries are critical in providing excellent patient-centre care and clinical research as well as basic science research. Registries evaluate current practice and guide future practice. The Flinders Kidney Health Registry will provide the key information needed to assess various treatment outcomes of patients with kidney cancer and patients who underwent nephrectomy for other reasons. The registry aims to provide clinical decision makers with longitudinal data on patient outcomes, health systems performance, and the effect of evolving clinical practice. The registry will also provide a platform for large-scale prospective clinical studies and research.

**Methods:**

Patients above the age of 18 undergoing nephrectomy or radiofrequency ablation for any indication and patients with SRMs will be included in the registry. Demographic, clinical and quality of life data will be collected from hospital information systems and directly from the patient and/or caregiver.

**Discussion:**

The Registry will report a summary of patient characteristics including indication for treatment, clinical risk profiles, surgical and oncological outcomes, the proportion of patients who progress to CKD and end stage kidney disease, quality of life post treatment as well as other relevant outcomes for all patients who have undergone nephrectomy for any indication, ablation or surveillance for SRMs. The registry will record the follow-up practice after nephrectomy and patient on active surveillance, which will help to develop and enhance a best practice protocol. The collected prospective data will provide a platform for ongoing patient-orientated research and improve patient-centred healthcare delivery.

## Background

Kidney cancer accounts for about 2% of malignant diseases in adults with an increasing worldwide incidence of over 430,000 new cases and 170,000 deaths per year [1]. It is one of the ten most common forms of cancer in developed countries and the aetiology is not well understood [2]. It often develops without symptoms, and therefore diagnosed incidentally.

Kidney cancer is not a single disease but consists of a number of different cancers, each with a different histology and different clinical course. Approximately 90% of the kidney cancers are renal cell carcinomas (RCC) and the remaining 10% of kidney cancers are transitional cell carcinomas (TCC) and others. RCC has multiple subtypes including clear cell RCCs (ccRCC), papillary RCCs (pRCC), and chromophobe RCCs (crRCC).

Radical nephrectomy or partial nephrectomy remains a cornerstone of the treatment of localised RCCs. In the past, survival times for patient with advanced or metastatic RCC rarely exceeded one year. However, there is trend towards earlier stage diagnosis with incidental imaging findings. Furthermore, there has been an explosion of therapies, including targeted therapies against angiogenesis and immunotherapy that target immune checkpoints such as programmed cell death–1 (PD-1) in the past ten years [3] which have led to improved survival outcomes.

Open or laparoscopic radical nephrectomy is the recommended treatment for stage T1b-T4 RCC. Nephron-sparing surgery or partial nephrectomy is the preferred treatment for smaller renal cancers (< 5 cm), which has equivalent oncologic outcomes and better renal function outcomes compared with radical nephrectomy. This is important in patients with a solitary kidney, bilateral renal cancers or pre-existing significant renal impairment [4]. The role of cytoreductive nephrectomy in patients with advanced or metastatic RCC is controversial but may be beneficial for selected patients despite the success of systemic therapy options.

Radical or partial nephrectomy for RCC is a risk factor for both new-onset and deterioration of existing chronic kidney disease (CKD) [5–9]. In contrast, nephrectomy in adult kidney donors with a normal contralateral kidney has shown little risk of new-onset CKD, although there are renal and cardiovascular effects with nephrectomy even in living kidney donors [10–13]. The reason for this difference in outcomes is not yet clearly understood. However, given the increasing prevalence of RCC [14], understanding risk factors for new-onset or deterioration of existing CKD after nephrectomy is important in developing preventive measures to provide better care for these patients. In addition to the risk of progression to end-stage kidney disease (ESKD), CKD is a major risk factor for cardiovascular disease and is associated with an increased risk of morbidities, hospitalisation, and mortality from any cause. Due to its increasing prevalence, associated with increased morbidity, mortality and health care cost, CKD is a major global health challenge.

Prospective data is required to examine the long-term impact of nephrectomy on the development of CKD and ESKD and the potential bidirectional and causal relationship between RCC and CKD. More studies are needed to explore the pathophysiology of developing CKD after nephrectomy, especially the role of compensatory renal hypertrophy, and to quantify the perioperative risks, and the role of other factors at a disease, patient, health care provider, institute and socio-demographic level in determining patient outcomes.

Small renal masses (SRMs) are increasingly diagnosed incidentally during the investigation of other medical conditions with abdominal ultrasound or CT scan. This has caused management challenge because many SRMs are either benign tumours such as oncocytoma or are renal cancers with relatively indolent behaviour [15]. There has been increased utility of percutaneous biopsy for SRMs as it can change the management plan, lower treatment costs and improve patient’s quality of life. Percutaneous biopsy for SRMs is safe, the risk of bleeding and needle track seeding is very low [16]. Partial and radical nephrectomy provide good oncologic control for small RCCs but are associated with development of new onset CKD or worsening of pre-existing CKD and increased cardiovascular morbidity [7]. Therefore, ablative therapies or active surveillance may be appropriate for SRMs due to RCC. The value of treating SRMs (< 4 cm) is questionable, especially in elderly patients with multiple comorbidities, given that most are benign or slowly growing; and patients are typically asymptomatic. There is a need to understand the incidence, natural history, management trends, sequelae of treatment, renal function and surveillance of SRMs. It is important that these patients are captured and participate in the registry. There is increasing evidence that there has been overtreatment of Bosniak 3 classified cystic lesions in the past [17, 18] and this will be examined in the Flinders Kidney Health Registry.

Clinical quality registries play an increasingly significant role in modern evident based clinical practice and research. Currently, there is no nephrectomy registry in Australia. The Flinders Kidney Health Registry within the Southern Adelaide Local Health Network (SALHN) will provide the key information needed to assess various treatment outcomes of patients with kidney cancer or patients who underwent for nephrectomy for any indication. The Registry aims to provide clinical decision makers with longitudinal data in the real-world setting on patient outcomes, health systems performance, health-care usage, the effect of evolving clinical practice, risk stratification based on patient risk characteristics and practice guideline development and improvement. Data are needed for development of protocols and guidelines. This registry will also provide a platform for large-scale prospective patient-orientated clinical studies and research.

## Methods and design

### Aims

The Registry represents both a clinical quality initiative within Departments of Urology and Renal Medicine in Southern Adelaide Local Health Network (SALHN) (Primary Aims), and a clinical research activity (Secondary Aims).

The primary aim of the Registry is to monitor patient outcomes, health system performance, health-care usage and the effect of evolving clinical practice within the Department of Urology and Department of Renal Medicine in SALHN. Specifically, the Registry aims to evaluate:

1. Long-term oncological and non-oncological outcomes after radical and partial nephrectomy for kidney cancers
2. Long-term outcomes after radical and partial nephrectomy for non-neoplastic kidney disease indications
3. Long-term kidney and non-kidney outcomes after nephrectomy for living kidney transplant donation
4. Long-term outcomes of patients with kidney cancer treated with ablative therapies
5. Long-term clinical outcomes of patients with SRMs who receive active surveillance
6. Quality of life post nephrectomy

The secondary aims of the Registry are to examine the prevalence, extent and pathophysiology of compensatory renal hypertrophy of the remaining kidney, new-onset or deterioration of existing CKD and the efficacy of different treatment modalities in post nephrectomy care. Specifically, the secondary aims are to evaluate:

1. Risk factors for new-onset CKD or deterioration of pre-existing CKD after nephrectomy for kidney cancers or non-neoplastic kidney indications
2. The clinical significance of post-nephrectomy acute kidney injury (AKI) on long-term outcomes
3. The incidence and effects of coexistent non-neoplastic histopathology changes and score in the nephrectomised kidney on long-term kidney function and other outcomes
4. Compensatory renal hypertrophy in the contralateral kidney after radical and partial nephrectomy
5. Comparison of oncological and non-oncological outcomes of different treatment modalities (radical nephrectomy vs partial nephrectomy vs radiofrequency ablation) for small localized renal malignancies
6. Establishment of a clinical risk score to predict the risk and clinical outcomes post nephrectomy which can be used in clinical consultation with patients before nephrectomy and guide the post nephrectomy follow up and management

Flinders Medical Centre (FMC) is a 750-bed tertiary referral teaching hospital accepting about 55,000 admissions per year. It provides medical services for nearly 350,000 people living in the southern metropolitan area of Adelaide, South Australia. We perform about 80 nephrectomy procedures per year. We also have retrospective data on 680 nephrectomy cases over the past 10 years.

### Case ascertainment

Inclusion criteria include:

- Patients undergoing radical nephrectomy or partial nephrectomy at SALHN for any indication
- Aged > 18 years at the time of nephrectomy

Patients with SMRs managed with radiofrequency ablation and/or active surveillance are also eligible providing that they meet the following criteria:

- Any T1 tumours (size up to 10 cm)
- Tumours can be solid or cystic (Bosniak 2F, 3 or 4)
- Referred to Department of Urology in SALHN
- Biopsy is not necessary for inclusion in the active surveillance
- Must have a least two cross sectional imaging encounters

### Consent

An opt-out consent model will be employed to ensure this registry can function effectively and maintain quality. The data on outcomes generated by this registry is likely to be compromised if the participation rate is not near complete, and the requirement for explicit consent would compromise the necessary level of participation. Involvement in this registry carries minimal risk.

In order to satisfy the requirement for opt-out consent, this registry will.

- Provide prospective participants with written plain language information explaining the nature of the information to be collected, the purpose of collecting it, the confidentiality and the simple procedure to decline participation
- Allow a reasonable time period between the participant receiving information and the collection of their data so that they have opportunity to withdraw
- Allow participants to opt-out of the registry at any time without any effect on their clinical care and without cost
- Develop a website which will provide participants further information and register their intension for non-participation

While participants can opt-out at any time, after a period of two weeks has elapsed patient data will be collected into the registry. If participants opt-out after this two-week period they can nominate to have all clinical details removed or simply not receive any follow up calls.

### Abstracting cases

The Registry Officer will manually collect data on Registry case report forms (CRFs) from hospital medical records, electronic health information systems and directly from the patient or patients’ family/caregiver, as appropriate.

### Summary of the data to be collected

#### Demographic information

- Patient identifiers: Unique Unit Record Number (URN), name, gender, date of birth, ethnicity
- Contact details: Address, email
- Physical characteristics: Height, weight and body mass index (BMI)
- Funding: Medicare number, category of funding (for health data linkage purpose)
- Comorbidities: Charlson comorbidities, Charlson score, American Society of Anaesthesiologists (ASA) grade, other comorbidities, smoking status
- Medications: Including all medications especially antihypertensive medications, angiotensin-converting enzyme (ACE) inhibitor, angiotensin receptor blocker (ARB), sodium‑glucose co‑transporter 2 (SGLT2) inhibitors antiplatelet agents, lipid lowering medications, perioperative antibiotics usage

#### Pre-surgical assessments (including nephrectomy or partial nephrectomy patients)

- Preoperative presentation and diagnosis
- Biopsy confirmation and details if available
- Staging investigation
- Laboratory investigations: full blood count (FBC), biochemistry results including serum creatinine, estimated glomerular filtration rate (eGFR), liver function test results, urinary albumin-to-creatinine ratio (ACR) or urinary protein-to-creatinine ratio (PCR)

#### Surgical information

- Procedure details: type of surgery, technique used, lymph node dissection, explorations of vena cava, blood loss, ischemic time, operative time, surgeon involved
- Post-operative course: post-operative complications, need for Intensive Care Unit (ICU) admission, length of hospital stays, readmission
- Pathology report including type of cancer, tumour diameter, number of tumours, margin, stage of cancer, grade, presence of necrosis, sarcomatoid transformation, presence of invasion, growth pattern, Fuhrman score, International Society of Urologic Pathologists (ISUP) score, non-neoplastic pathology includes: diabetic nephropathy, hypertensive changes, glomerulonephritis, global and segmental glomerulosclerosis (GS), tubular atrophy (TA), interstitial fibrosis (IF) and arteriosclerosis

#### Post-surgical follow-up assessments

- Follow-up assessments such as date of follow-up, patient status, laboratory & radiology reports, presence of recurrence, need for dialysis
- Serial measurements of serum creatinine, eGFR, proteinuria and haemoglobin
- Serial images in detection of recurrence or metastases, remaining kidney size and volume and evidence of compensatory renal hypertrophy

The time for follow up visits, blood tests, medical images and data collection points are summarised in Table 1. There will be no medical images after 60 months post nephrectomy unless clinically indicated. In term of blood tests, they will be collected annually after 60 months.

**Table 1:** Data collection times post nephrectomy

Post nephrectomy months	3	6	12	18	24	30	36	48	60
**Low risk/T1**									
Hx, PE, blood tests	√		√		√		√		√
Chest			CT		CXR		CT	CXR	CT
Abdomen	CT		CT		US		CT		CT
**Intermediate risk/T2**									
Hx, PE, blood tests	√	√	√	√	√	√	√	√	√
Chest		CXR	CT	CXR	CT	CXR	CT	CXR	CT
Abdomen	CT	US	CT	US	CT	US	CT	US	CT
**High risk/T2**									
Hx, PE, blood tests	√	√	√	√	√	√	√	√	√
Chest		CXR	CT	CXR	CT	CXR	CT	CXR	CT
Abdomen	CT	CT	CT	CT	CT	CT	CT	CT	CT

Hx: History, PE: Physical examination, CT: Computerized tomography, CXR: Chest X-ray, US: Ultrasound

#### Non-surgical management (including active surveillance and/or radiofrequency ablation)

- Data on surveillance such as date commenced, follow up information and need for treatment conversion
- Ablation treatment data such as date of ablation, subsequent ablation, follow up data
- Radiotherapy details
- Systemic therapy details such as type and duration of therapy, complications, response to systemic therapy

#### Quality of life (QoL)

Validated instruments (AQoL-6D and NCCN-FACT FKSI) will be used to assess quality of life. Patient Reported Outcome Measures will be requested at baseline (before treatment) and then at 12 and 24 months after diagnosis.

### Data linkage

The patient information sheet makes provision for the following data linkage activities.

- Death Registry. This will provide the date and cause of death for patients in the registry. Deaths captured by the South Australian Births Deaths and Marriages Registry will be linked
- Medicare Benefits Schedule (MBS) and Pharmaceutical Benefits Scheme (PBS). These two data sources will provide greater definition around medications and procedures relating to each participant.

### Quality assurance

The Registry will be subject to ongoing quality assurance audits. Annually, 10% of eligible patient records will be reviewed to ensure clinical and outcomes data abstracted by the Registry Officer remain consistent with the clinical record. The Quality Assurance (QA) Plan, acceptable discrepancy rates and remedial actions will be detailed in an appropriate standard operating procedure.

### Definition of outcomes

#### Tumour stage

Tumour stage will be determined according to the tumour, nodes and metastases system (TNM) and the cellular grade as grades 1–4 using the Fuhrman system.

#### Co-existing non-neoplastic kidney disease

Detailed examination of glomerular, tubular, interstitial and vascular pathology will be performed on all nephrectomy specimens by SA pathology service in accordance with the Royal Australasian College of Pathologist guidelines [19]. The co-existing non neoplastic kidney disease (NNKD) will be classified into five categories: (i) Hypertensive-related changes, (ii) Diabetes-related changes, (iii) Combination of hypertensive and diabetes-related changes, (iv) Glomerulonephritis and (v) Other non-specific changes. Global and segmental glomerulosclerosis (GS), tubular atrophy (TA) and interstitial fibrosis (IF), and arteriosclerosis/arteriolosclerosis will be assessed and scored.

#### eGFR

The estimated glomerular filtration rate (eGFR) is calculated according to the Chronic Kidney Disease Epidemiology Collaboration (CKD-EPI) study equation [20]. The unit of measure is mL/min/1.73m^2^.

#### New onset CKD

The development of new onset CKD is determined by an eGFR < 60 mL/min/1.73m^2^ which developed after nephrectomy and persists for > 3 months, regardless of aetiology. The time to development of CKD after nephrectomy is defined as the date of nephrectomy to the date of the first eGFR < 60 mL/min/1.73m^2^.

#### Acute kidney injury

Acute kidney injury (AKI) is defined based on Kidney Disease: Improving Global Outcomes (KDIGO) guideline:

1. Increase in serum creatinine > 30 µmol/L within 48 h
2. Increase in serum creatinine > 1.5 times baseline in 7 days
3. Urine volume < 0.5 ml/kg/hour for 6 h

AKI is assessed by comparing the highest serum creatinine level within 7 days post-nephrectomy with the pre-operative serum creatinine level.

### Reporting

The Registry will report annually and provide data on the following metrics:

- the number and proportions of patients undergoing partial and radical nephrectomy
- summary of patient characteristics including indication for nephrectomy and clinical risk profiles
- the oncological outcomes
- the proportion of patients who progress to CKD and ESKD
- complication rate
- in hospital, 30 day and long-term mortality
- length of hospital stays, 7 day and 30 day readmission rate
- Quality of life at 12 months post treatment
- the outcomes of patients underwent active surveillance

## Discussion

Clinical outcomes monitoring is critical in ensuring that the patients are receiving the highest quality of care and is the key component of evidence-based practice. This requires high quality, prospective data. There is little literature in terms of perioperative and longer-term outcomes on kidney cancer treated with partial or radical nephrectomy or other modalities such as active surveillance or radiofrequency ablation within Australia. Furthermore, there is no registry data to monitor the clinical outcomes of nephrectomy for renal cancer or other indications to evaluate the current practice and improve the best practice guideline.

Best practice guidelines are essential feature of good clinical care. Guideline development requires access to patterns of care, quality of care, and a platform for research [21, 22]. Assessing practice guidelines will need data on clinical outcomes and adherence to recommended investigations, treatment and follow-up. Whilst controversy exists over whether early diagnosis of metastatic disease leads to an improved survival over late diagnosis, there is evidence that patients who undergo a structured, risk stratified follow up regime have a better prognosis than those who are not part of a follow up program [23]. All major follow up protocols for RCC address three main issues: relevant oncological information, functional information and psychosocial issues relating to survivorship and treatment. Treatment for RCC especially radical nephrectomy places patients at a higher risk for developing CKD or progressive pre-existing CKD. The functional aspect of follow up allows early detection of renal impairment as well as modifying risk factors such as hypertension, better control of diabetes, and dyslipidaemia. Although there are international post nephrectomy for RCC follow-up protocols, no Australian follow-up guidelines currently exist [24–26]. We have developed a local SALHN follow-up protocol post nephrectomy for renal cancers (Table 1). This registry would aid in the monitoring of local follow-up practice and this will help to develop and refine national follow-up protocols for patients treated with nephrectomy or active surveillance or radiofrequency ablation therapies.

There is no evidence regarding the best management strategies of SRMs (confirmed or suspicious for renal cancer) in patient with pre-existing CKD or other comorbidities. Active surveillance is increasingly accepted as a treatment modality. This is particularly true of the elderly who may not be fit for surgery or dialysis. It is important to collect data prospectively to monitor the progression of a mass, oncological outcomes and trends in renal function. Immunotherapy has significantly changed the treatment landscape for patients with advanced or metastatic RCC. A clinical quality registry, with its data collection, feedback and practice improvement loop is the ideal tool for monitoring and improving guidelines relating to nephrectomy in Australia; as well as providing a basis for research studies [27].

Health related quality of life (HRQoL) measures are increasingly being recognised as important determinants of treatment outcomes. HRQoL measures include patient reported symptoms, assessment of general function including satisfaction or dissatisfaction with areas that are deemed important to the patient. HRQoL measures provide information about the impact of disease processes and treatments to individual patients, the health care systems and to society.

Patients with localised kidney cancer usually enjoy an excellent prognosis. Recently there has been increased interest in this cohort of patients to measure HRQoL outcomes as treatments for kidney cancer like nephrectomy can reduce quality of life for many years subsequent to the treatment exposure especially if the patient develops advanced CKD or ESKD requiring renal replacement therapy such as haemodialysis.

There have been a number of studies examining patient reported outcome measures (PROMs) and quality of life in kidney cancer [28]. The European Organisation for Research and Treatment of Cancer (EORTC) has developed and tested the first HRQoL questionnaire specific to renal cell cancer (EORTC-QLQ- C30) [23]. To date there has been no publications that have focused on health-related quality of life outcomes on Australian patients with kidney cancer. The Flinders Kidney Health Registry and the proposed prospective collection of HRQoL measures using the EORTC-QLQ-C30 are ideally placed to report the first HRQoL outcomes specific to patients with kidney cancer in Australia. This will help clinicians to improve the care of these patients.

There are several limitations for this registry: (a) These routinely collected data points are not for a specific research project so their timing, quality and completeness may not meet all needs; (b) The catchment area for the registry is local rather than national at this stage and (c) This registry does not enrol and collect data from patients who are managed in the private hospital and private practice. Despite these limitations, this protocol describes a unique registry providing a valuable quality improvement and research resource.

## Data Availability

Data requests will be considered on a case-by-case basis.

## Abbreviations

AKI: Acute kidney injury
CKD: Chronic kidney disease
CRF: Case report form
DVA: Department of veterans’ affairs
eGFR: Estimated glomerular filtration rate
ESKD: End-stage kidney disease
FMC: Flinders medical centre
HREC: Human research ethics committee
MBS: Medicare benefits schedule
NHS: Noarlunga health service
NNKD: Non-neoplastic kidney disease
PBS: Pharmaceutical benefits schedule
QA: Quality assurance
QoL: Quality of life
RCC: Renal cell carcinoma
SALHN: Southern Adelaide local health network
SRMs: Small renal masses
